# Prevalence of dry eye during the COVID-19 pandemic: A systematic review and meta-analysis

**DOI:** 10.1371/journal.pone.0288523

**Published:** 2023-12-13

**Authors:** Haiyang Ji, Yun Yang, Yunqiong Lu, Xiehe Kong, Guang Yang, Jie Liu, Yanting Yang, Xuejun Wang, Xiaopeng Ma

**Affiliations:** 1 Yueyang Hospital of Integrated Traditional Chinese and Western Medicine, Shanghai University of Traditional Chinese Medicine, Shanghai, China; 2 Shanghai Research Institute of Acupuncture and Meridian, Shanghai, China; 3 Eye & ENT Hospital, Fudan University, Shanghai, China; University of New South Wales, AUSTRALIA

## Abstract

**Objective:**

During the COVID-19 pandemic, many people devoted longer time to screen viewing due to the need for study, work, and online social activities, instead of outdoor activities, which may have led to an increase in dry eye symptoms. This study aimed to evaluate the prevalence of dry eye during the COVID-19 pandemic.

**Methods:**

PubMed, Cochrane Library, Embase, and Web of Science were searched from January 1, 2020 to October 20, 2022. Cross-sectional surveys on dry eye prevalence conducted after January 1, 2020 were included. Two review authors independently performed data extraction and assessed study quality. The random-effects model was used to analyze the prevalence of dry eye, and the odds ratio was used to assess the strength of the association between variables. Subgroup analysis was performed to detect heterogeneity, the leave-one-out method for sensitivity analysis, and the Egger test for publication bias.

**Results:**

A total of eleven studies with 15692 individuals met the eligibility criteria. The prevalence of dry eye during the COVID-19 pandemic was 61.0% (95%CI: 51.8%-70.2%) globally and 56.7% (95%CI: 45.3%-68.1%) in Asia. The prevalence of dry eye had significant differences in sex and visual display time, with higher prevalence among females and visual display time of more than 4 hours per day. Subgroup analysis was performed based on diagnostic tools, study population, and average age. A significant difference was found in diagnostic tools, but no significant change in heterogeneity (*P*<0.05). The leave-one-out method showed stable results, and the Egger test identified no significant publication bias.

**Conclusion:**

The prevalence of dry eye during the COVID-19 pandemic is significantly higher than before, and a higher prevalence is found among females and those having a visual display time of more than 4 hours per day.

## Introduction

During the latter part of 2019, COVID-19 struck the world and significantly changed people’s lifestyles: people reduced outdoor activities and spent more time on screens due to the lockdown measures [1]. Recent epidemiological surveys have shown an increase in the prevalence of psychological disorders such as anxiety and depression during the COVID-19 pandemic [2, 3], as well as a significant increase in the number of people suffering from digital eye strain due to the change of lifestyle [4–6].

It is well known that the prevalence of dry eye is associated with the use of video display units [7–9]. Dry eye is characterized by a loss of the tear film homeostasis, accompanied by ocular symptoms including dryness, discomfort, pain, grittiness, blurry vision, foreign-body sensation, and visual disturbance [10]. Not only does dry eye affect people’s quality of life and reduce their work efficiency [11–13], but it also increases the economic burden on society [14].

For providing evidence for policy development and health care, it is essential to investigate the prevalence of dry eye during the COVID-19 pandemic. This study is the first systematic review and meta-analysis on the prevalence of dry eye during the COVID-19 pandemic.

## Methods

### Registration

This study was registered on PROSPERO (CRD42022369997) and conducted based on the Preferred Reporting Items for Systematic Reviews and Meta-Analyses (PRISMA) statement guidelines [15].

### Ethics statement

This study does not require formal ethical approval, given that all data collected in this study will not contain individual patient data, and there will be no concerns about privacy.

### Search strategy

PubMed, Cochrane Library, Embase, and Web of Science were retrieved for relevant studies published from January 1, 2020 to October 20, 2022. The search terms are as follows: (“Dry Eye Syndrome” OR “Dry Eye” OR “Dry Eye Disease” OR “Evaporative Dry Eye Disease”) AND (“COVID-19” OR “SARS-CoV-2 Infection” OR “2019 Novel Coronavirus Disease” OR “2019 Novel Coronavirus Infection” OR “2019-nCoV Disease” OR “COVID-19 Virus Infection” OR “Coronavirus Disease 2019”).

### Inclusion criteria

1. Cross-sectional survey based on the general population.
2. Studies conducted after January 1, 2020.
3. Prevalence rates can be obtained directly or calculated from relevant data.
4. The language is limited to English.

### Exclusion criteria

1. Epidemiological studies conducted in eye clinics or based on dry eye patients.
2. The number of participants in the survey is smaller than 30.
3. The conference abstracts with relevant data.

### Study selection and data extraction

Two researchers independently searched all databases to identify eligible studies based on inclusion and exclusion criteria and extracted data including first author, publication year, country or region, sample size, diagnostic tools, study population, age, sex composition, and prevalence. If the information was incomplete, we contacted the author(s) by e-mail. Any disagreement was resolved through discussion with a third reviewer.

### Quality assessment

Two researchers independently assessed the quality of studies included in the final analysis using the Joanna Briggs Institute Quality Assessment Tool [16]. According to the criteria, each item will be classified as “Yes”, “No”, “Unclear”, and “Not applicable”. Any disagreement was resolved through discussion with a third researcher.

### Statistical analysis and publication bias

The prevalence of dry eye during the COVID-19 pandemic was analyzed with Stata 16 software (Stata Corp LP, College Station, TX, USA), and Odds ratio (OR) was calculated with Review Manager 5.4 (Nordic Cochrane Center, The Cochrane Collaboration, Copenhagen, Denmark). *I*^2^>50% was considered significantly heterogeneous. As the included studies differed in average age, study population, diagnostic tools, and time of study execution, a random-effects model were used to make our results more stable. Subgroup analysis was performed based on average age, study population, diagnostic tools, and time of study execution to detect sources of heterogeneity and to analyze the results of populations with different characteristics. The leave-one-out sensitivity analysis was performed to evaluate results and the Egger test for publication bias.

## Results

### Literature search results and basic characteristics

**Fig 1:**
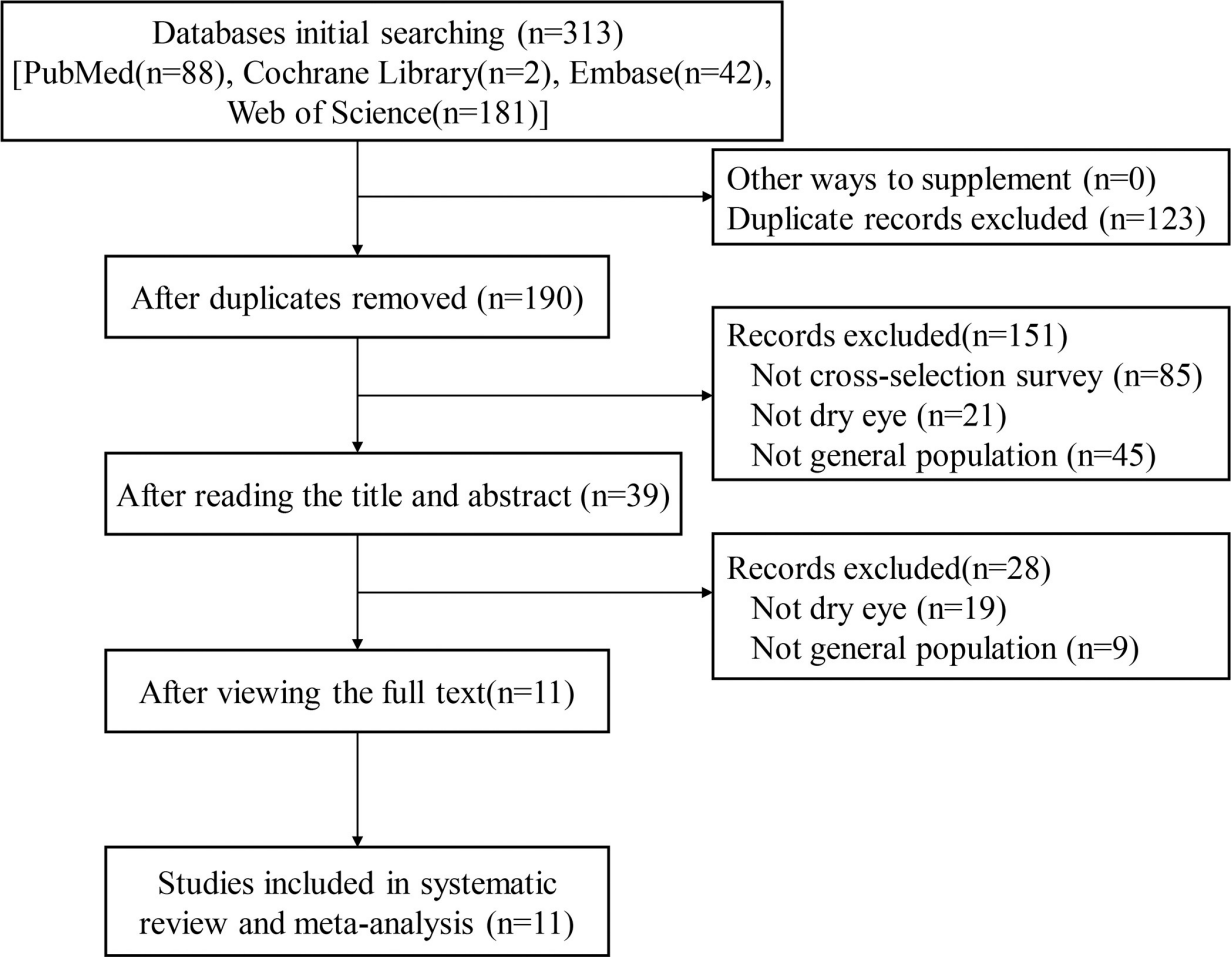
Flow chart of literature retrieval. Flow diagram for including eligible studies in the meta-analysis.

By searching the relevant databases, we retrieved a total of 313 articles [PubMed (n = 88), Cochrane Library (n = 2), Embase (n = 42), Web of Science (n = 181)], and 11 met the criteria [17–27] (Fig 1).

Eleven studies with 15692 participants were conducted in eight countries and regions [17–27]. Most of these studies were published in 2022 [17–24, 26], and the study populations included school children [18, 22, 27], university students [19, 21, 23, 24, 26], healthcare workers [17, 25], and other professionals [20]. All studies used various dry eye questionnaires as diagnostic tools (Table 1), and the quality assessment (Table 2) suggested that the studies were of high quality.

**Table 1:** The characteristics of studies included in the systematic review and meta-analysis.

Study	Country	Time of study execution	Sample size (n)	Sex (n) Male / Female	Age [Min-Max (-x±s)]	Study population	Prevalence (%)	Diagnostic tools
Allayed 2022	Palestine	2021.03–2021.10	300	174/126	23–55 c34.6±8.3)	Nurses	62.0	OSDI
Rao 2022	India	Unclear	3327	1675 /1520(132 unclear)	Unclear	Students and teachers	60.7	Unclear
García-Ayuso 2022	Spain	2020.10–2020.11	676	185/491	18-42(20.7±2.9)	University students	71.3	OSDI
Salinas-Toro 2022	Chile	2020.05–2020.07	1797	541/1256	(40.5±11.1)	Teleworking	67.7	DEQ-5
Abdulmannan 2022	Iraq, Jordan	2021.11–2022.01	1431	432/999	≥18	University students	29.0	VFH-25, WHS
Lin 2022	China	2021.11–2021.12	4694	2248/2446	14-19(16.39±0.95)	High school students	70.5	OSDI
Al-Dolat 2022	Jordan	2020.10–2020.11	1219	508/711	Unclear	Medical students	71.7	OSDI
Tangmonkongvoragul 2022	Thailand	2020.11–2021.01	528	252/276	17-31(20.48)	Medical students	70.8	OSDI
Long 2020	China	2020.01–2020.03	53	16/37	24-45(32.43)	Doctors and nurses	35.8	OSDI
Cartes 2022	Chile	2020.05	1450	508/942	17-46(21.1±2.7)	University students	77.4	DEQ -5
Mohan 2021	India	Unclear	217	101/116	10-18(13±2.45)	School children	50.2	CVS-Q

Min: Minimum; Max: Maximum; -x±s: Mean ± standard deviation; OSDI: Ocular Surface Disease Index; DEQ-5: Dry Eye Questionnaire 5, VFQ-25: Visual Functioning Questionnaire-25; WHS: Women’s Health Study Questionnaire; CVS‑Q: Computer Vision Syndrome Questionnaire.

**Table 2:** The quality assessment of studies included in the systematic review and meta-analysis.

Study	Q1	Q2	Q3	Q4	Q5	Q6	Q7	Q8	Q9	Q10
Allayed 2022	Y	Y	Y	Y	Y	Y	Y	Y	Y	Y
Rao 2022	Y	Y	U	Y	Y	N	Y	Y	Y	Y
García-Ayuso 2022	Y	Y	U	Y	Y	Y	Y	Y	Y	Y
Salinas-Toro 2022	Y	Y	U	Y	Y	Y	Y	Y	Y	Y
Abdulmannan 2022	Y	Y	U	Y	Y	Y	N	Y	Y	Y
Lin 2022	Y	Y	Y	Y	Y	Y	Y	Y	Y	Y
Al-Dolat 2022	Y	Y	U	Y	Y	Y	Y	Y	Y	Y
Tangmonkongvoragul 2022	Y	Y	U	Y	Y	Y	Y	Y	Y	Y
Long 2020	Y	Y	U	Y	Y	Y	Y	Y	Y	Y
Cartes 2022	Y	Y	U	Y	Y	Y	Y	Y	Y	Y
Mohan 2021	Y	Y	U	Y	Y	Y	Y	Y	Y	Y

The Joanna Briggs Institute Quality Assessment Tool

Q1: Was the sample representative of the target population?

Q2: Were study participants recruited in an appropriate way?

Q3: Was the sample size adequate?

Q4: Were the study subjects and the setting described in detail?

Q5: Was the data analysis conducted with sufficient coverage of the identified sample?

Q6: Were objective, standard criteria used for the measurement of the condition?

Q7: Was the condition measured reliably?

Q8: Was there appropriate statistical analysis?

Q9: Are all important confounding factors/subgroups/differences identified and accounted for?

Q10: Were subpopulations identified using objective criteria?

Y: Yes; N: No; U: Unclear; N/A: Not applicable.

### Prevalence of dry eye during the COVID-19 pandemic

**Fig 2:**
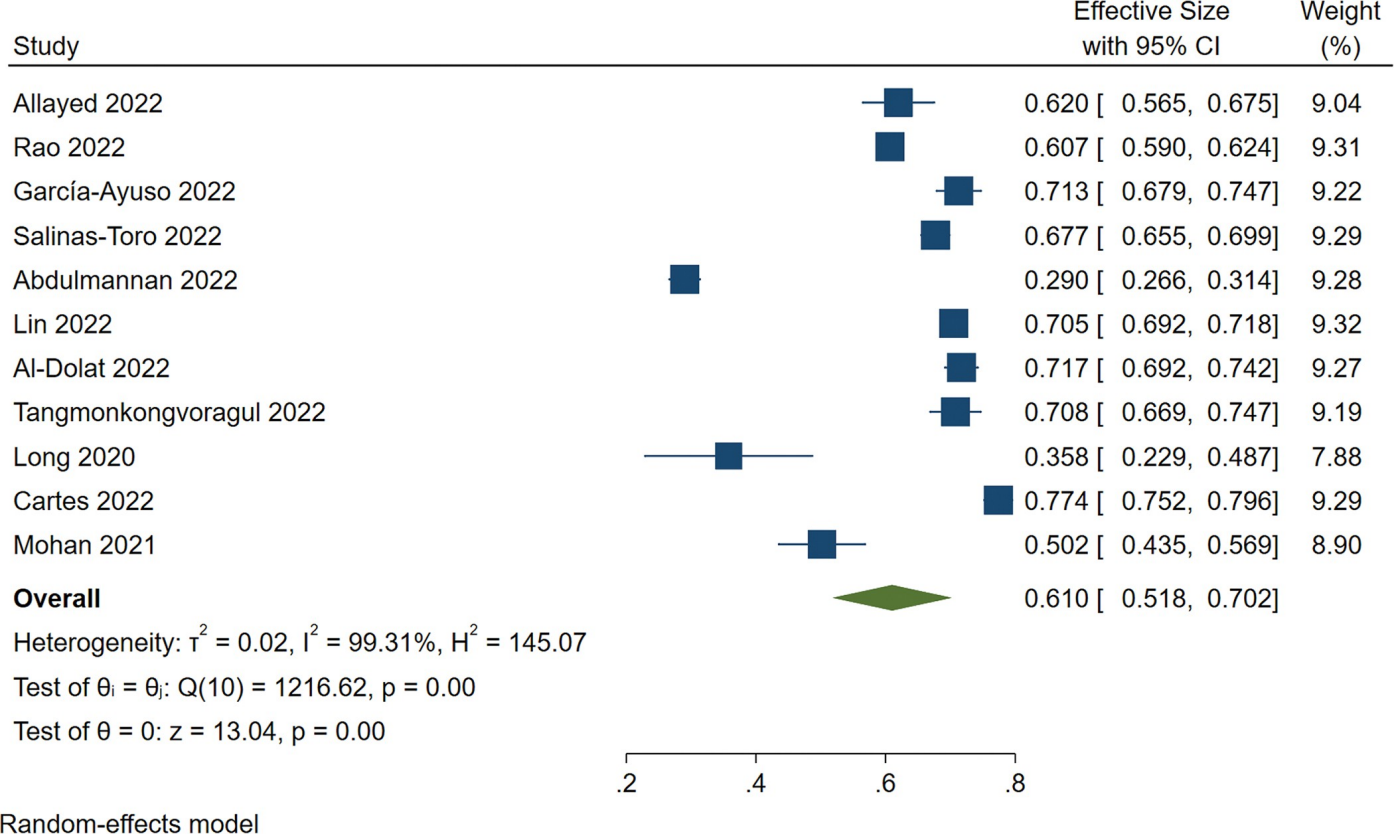
Forest plot of studies showing the overall prevalence of dry eye during the COVID-19 pandemic. The random-effects model was used. The pooled estimate was reported as prevalence as a percentage and CI. CI, Confidence interval.

**Fig 3:**
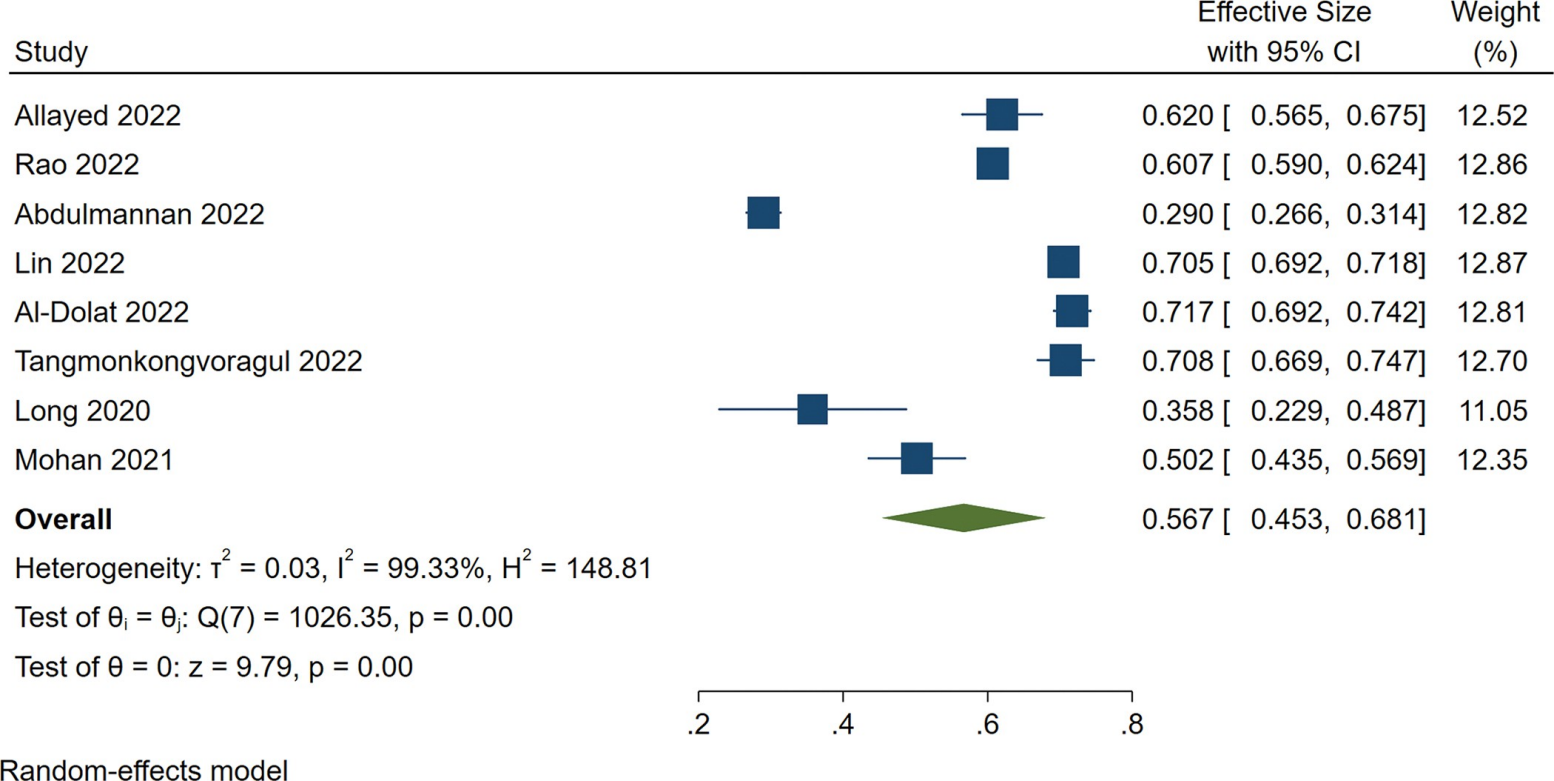
Forest plot of studies showing the prevalence of dry eye during the COVID-19 pandemic in Asia. The random-effects model was used. The pooled estimate was reported as prevalence as a percentage and CI. CI, Confidence interval.

The pooled estimate of dry eye prevalence during the COVID-19 pandemic was 61.0% (95%CI: 51.8%-70.2%), and there was significant heterogeneity among the studies (*I*^*2*^ = 99.31%, *P* = 0.00) (Fig 2). The prevalence of dry eye in eight of 11 studies conducted in Asia [17, 18, 21–25, 27] was 56.7% (95%CI: 45.3%-68.1%), and heterogeneity was also significant (*I*^*2*^ = 99.33%, *P* = 0.00) (Fig 3).

**Fig 4:**
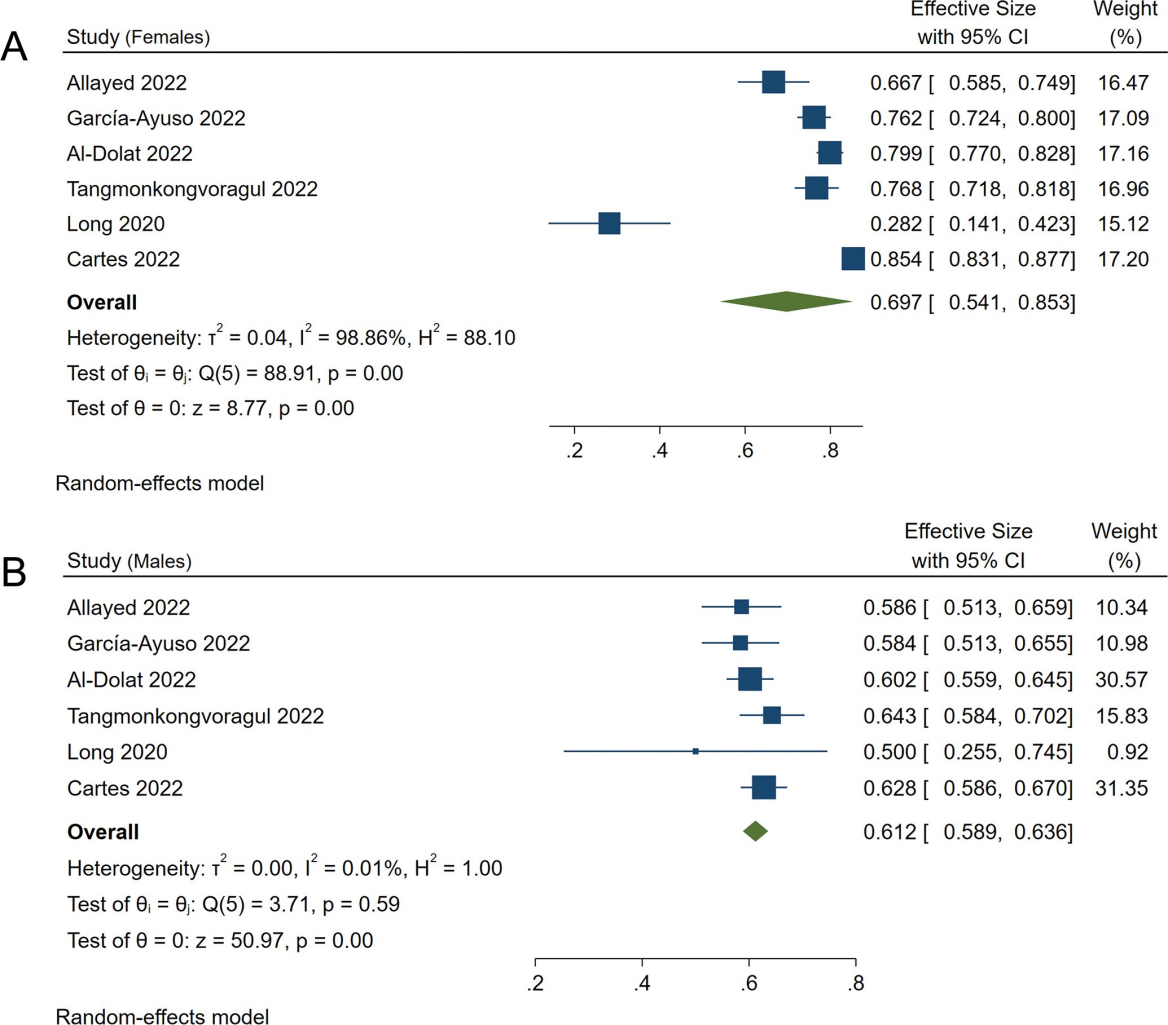
Forest plot of studies showing the prevalence of dry eye in females and males. The random-effects model was used. A and B refer to the prevalence of dry eye in females and males during the COVID-19 pandemic, respectively. The pooled estimate was reported as prevalence as a percentage and CI. CI, Confidence interval.

**Fig 5:**
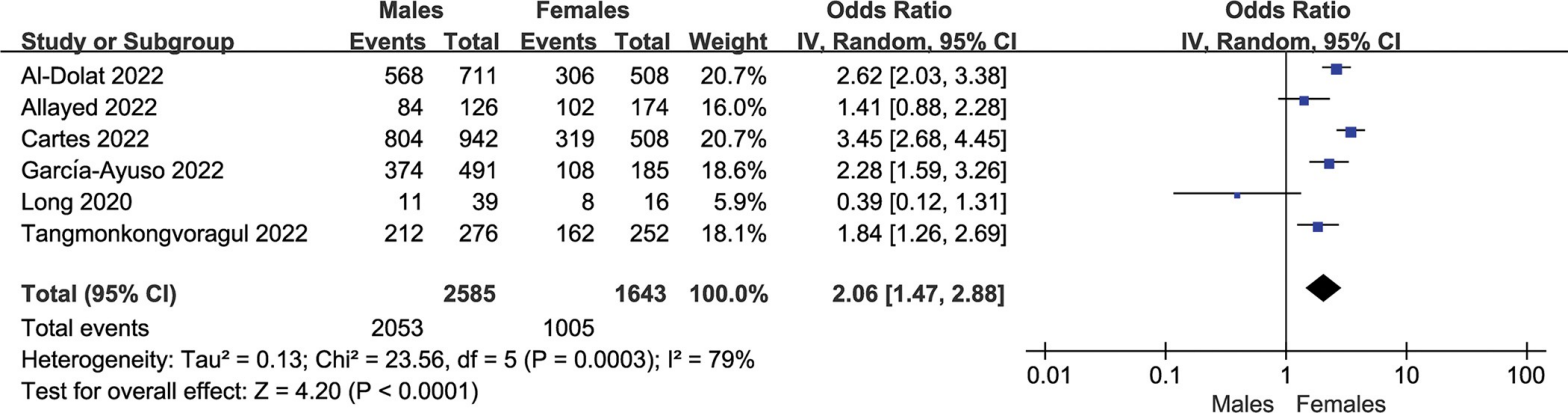
Forest plot of studies comparing the prevalence of dry eye between females and males. The random-effects model was used. The pooled estimate was reported as an odds ratio. CI, Confidence interval; IV, Inverse variance.

Six of the 11 studies reported the prevalence in males and females separately [17, 19, 23–26]. The prevalence of dry eye in females was 69.7% (95%CI: 54.1–85.3) (Fig 4A) and 61.2% in males (95%CI: 58.99%-63.6%) (Fig 4B). The OR was 2.06 (95%CI: 1.47–2.88), and there was a significant difference (*P<*0.001) (Fig 5).

Eight of the 11 studies reported the prevalence of dry eye in terms of severity [17–19, 22–25, 27]. The prevalence of mild dry eye was 23.9% (95%CI: 18.8%-28.9%) (S1A Fig), moderate was 16.4% (95%CI: 13.5%-19.3%) (S1B Fig), and severe was 22.0% (95%CI: 12.1%-31.9%) (S1C Fig).

Four studies reported the prevalence of dry eye in contact lens wearers and non-contact lens wearers [17, 19, 24, 26]. The prevalence of dry eye in contact lens wearers was 83.5% (95%CI: 75.7%-91.3%) (S2A Fig) and 68.2% in non-contact lens wearers (95%CI: 61.8%-71.4%) (S2B Fig). The OR was 1.90 (95%CI: 0.84–4.30) between contact lens wearers and non-contact lens wearers, and there was no significant difference (*P* = 0.12) (S3 Fig).

**Fig 6:**
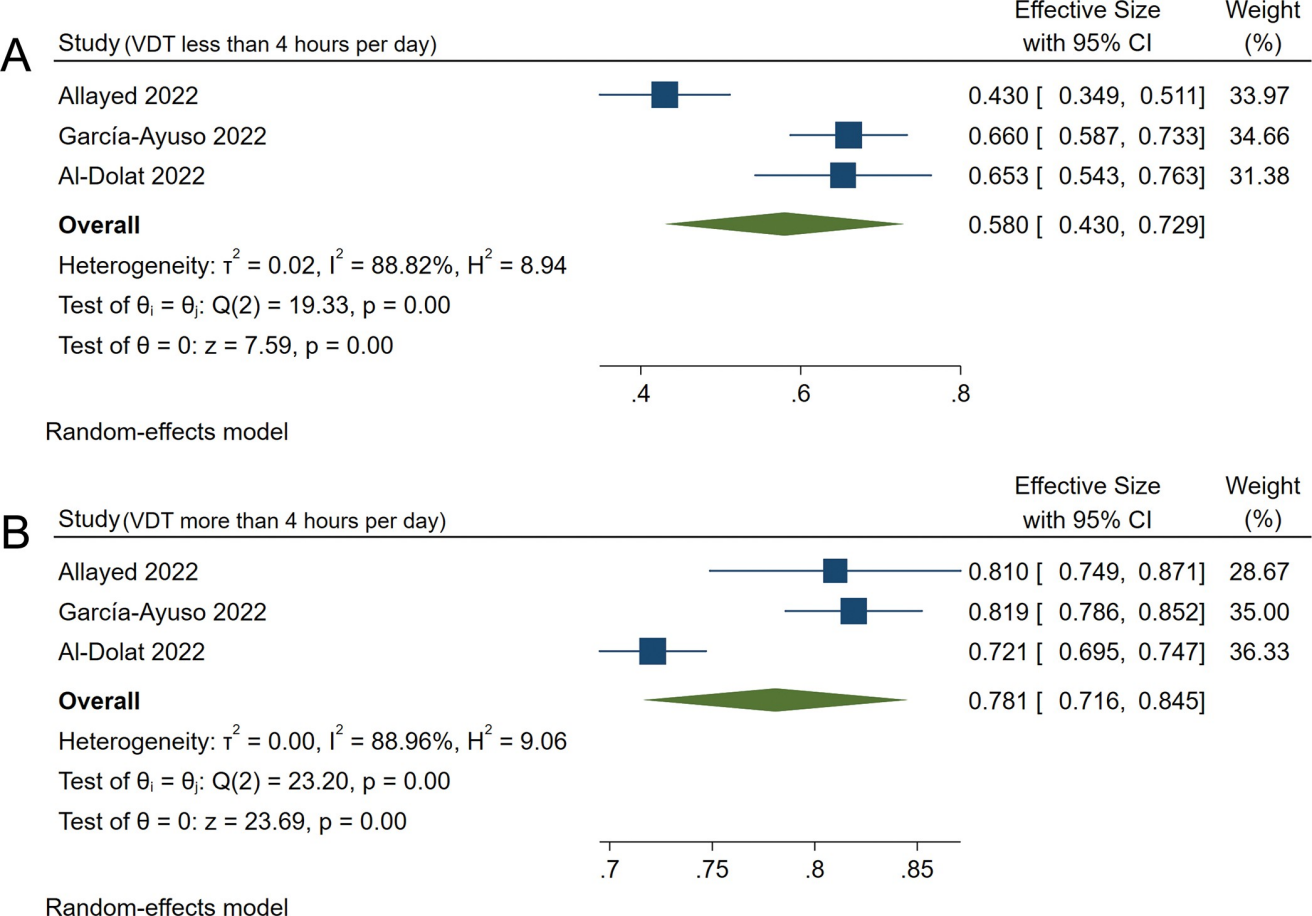
Forest plot of studies showing the prevalence of dry eye in VDT of less than and more than 4 hours per day. The random-effects model was used. A and B refer to the prevalence of dry eye during the COVID-19 pandemic in VDT of less than and more than 4 hours per day, respectively. The pooled estimate was reported as prevalence as a percentage and CI. VDT, visual display time; CI, Confidence interval.

**Fig 7:**
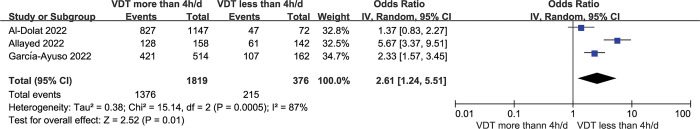
Forest plot of studies comparing the prevalence of dry eye between VDT of more than and less than 4 hours per day. The random-effects model was used. The pooled estimate was reported as an odds ratio. VDT, visual display time; CI, Confidence interval; IV, Inverse variance.

The prevalence of dry eye in visual display time (VDT) of less than 4 hours per day was 58.0% (95%CI: 43.0%-72.9%) (Fig 6A) and 78.1% in VDT of more than 4 hours per day was (95%CI: 71.6%-84.5%) (Fig 6B) [17, 19, 23]. The OR was 2.61 (95%CI: 1.24–5.51) between them, and there was a significant difference (*P* = 0.01) (Fig 7).

The prevalence of dry eye was 66.8% (95%CI: 21.4%-82.2%) among non-smokers (S4A Fig) and 74.0% (95%CI: 63.1%-85.0%) among smokers (S4B Fig) [17, 26]. The OR was 4.30 (95%CI: 0.69–26.72) between them, and there was no significant difference (*P* = 0.12) (S5 Fig).

### Subgroup analysis

#### Diagnostic tools

**Fig 8:**
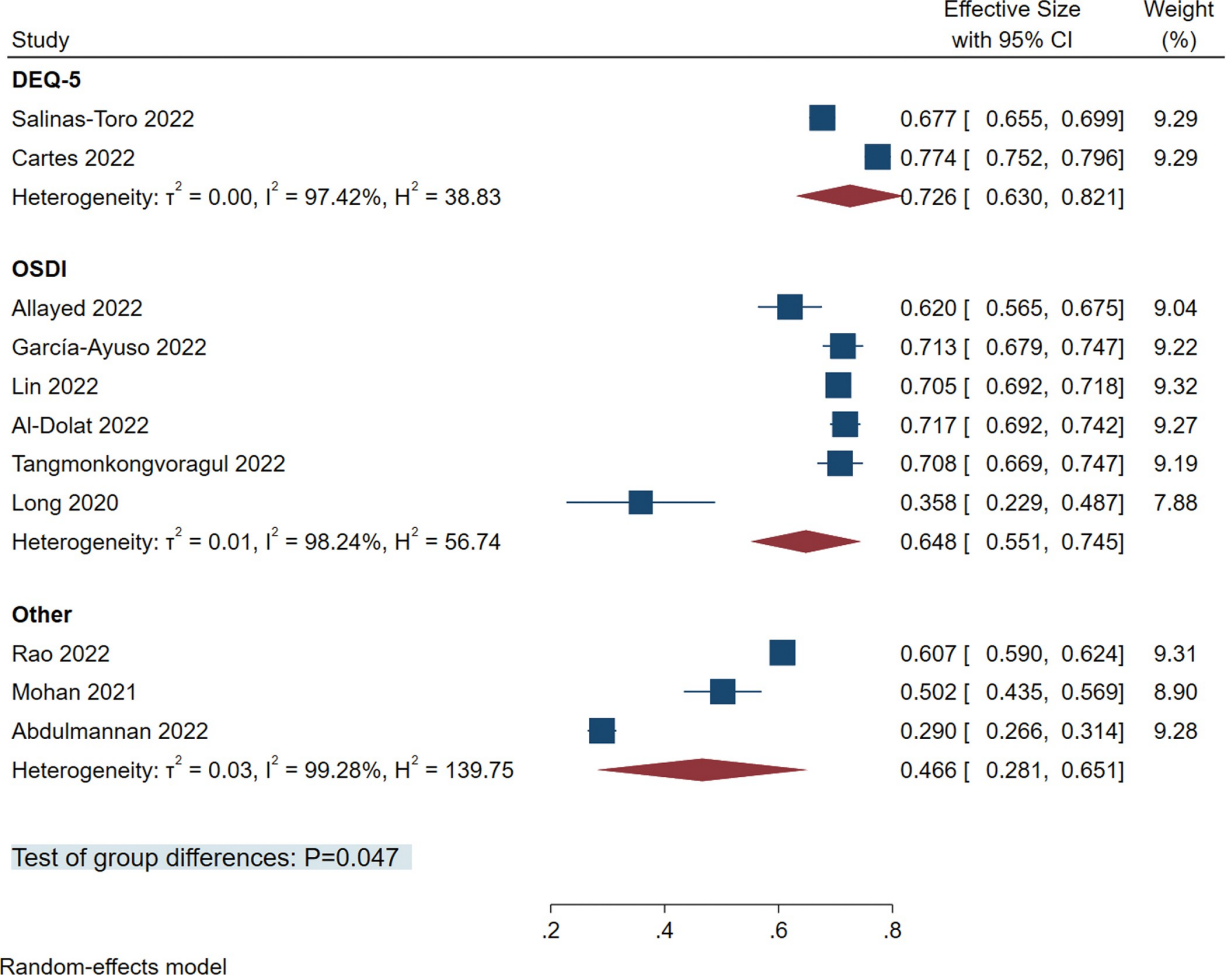
Forest plot of studies showing subgroup analysis of diagnostic tools. The random-effects model was used. The pooled estimate was reported as prevalence as a percentage and CI. OSDI, ocular surface disease index; DEQ-5, dry eye questionnaire 5; other tools included visual functioning questionnaire-25, women’s health study questionnaire, computer vision syndrome questionnaire; CI, Confidence interval.

The 11 studies [17–27] we included used different diagnostic tools for diagnosing dry eye in the study population (Table 1). In our subgroup analysis based on diagnostic tools, there were significant differences between the prevalence obtained using different diagnostic tools (*P* = 0.047): Ocular Surface Disease Index (OSDI), 64.8% (95%CI: 55.1%-74.5%); Dry eye questionnaire 5 (DEQ-5), 72.6% (95%CI: 63.0%-82.1%); and other tools, 46.6% (95%CI: 28.1%-65.1%0) (Fig 8).

#### Study population

There were no significant differences between study population (*P* = 0.826): school children, 60.8% (95%CI: 49.6%-72.1%); university students, 64.0% (95%CI: 46.7%-81.4%); and occupational population, 56.1% (95%CI: 37.7%-74.5%) (S7 Fig).

#### Average age

Subgroup analysis was performed based on average age, and a higher prevalence of dry eye was found in people over 30 years old (56.1%, 95%CI: 37.7%-74.5%), compared with 61.6% (95%CI: 46.8%-76.4%) in people under 30 years old. However, the difference was not significant (*P* = 0.649) (S7 Fig).

#### Time of study execution

Subgroup analysis of the studies before and after January 2021 was also performed: the prevalence of dry eye was 53.8% (95%CI: 28.9%-78.7%) before January 2021 and 66.8% (95%CI: 56.4%-77.2%) after January 2021, but there was no significant difference (*P* = 0.344) (S8 Fig).

#### Heterogeneity test

Subgroup analysis was performed to explore the sources of heterogeneity. No significant change was found in heterogeneity by performing subgroup analyses on mean age, diagnostic tools, and study population (Table 3).

**Table 3:** Heterogeneity test of subgroup analysis.

Subgroup		Studies (n)	Prevalence (%)	95%CI (%)	Heterogeneity across the studies		Heterogeneity between groups (*P* value)
					I^2^ (%)	*P*	
Average age	≥30 years old	3	56.1	37.7–74.5	97.00	0.00	0.00
	<30 years old	6	61.6	46.8–76.4	99.51	0.00	
Study population	School children	3	60.8	49.6–72.1	98.88	0.00	0.00
	University students	5	64.0	46.7–81.4	99.51	0.00	
	Occupational population	3	56.1	37.7–74.5	97.00	0.00	
Diagnostic tools	OSDI	6	64.8	55.1–74.5	97.42	0.00	0.00
	DEQ-5	2	72.6	63.0–82.1	97.42	0.00	
	Others	3	46.6	28.1–65.1	99.28	0.00	
Time of study execution	Before January 2021	3	53.8	28.9–78.7	99.67	0.00	0.00
	After January 2021	6	66.8	56.4–77.2	98.60	0.00	

CI: Confidence interval.

### Sensitivity analysis

Leave-one-out sensitivity analysis was also conducted to examine further the possible cause of heterogeneity across the studies involved in the analysis and test the stability of the result. With this analysis, no significant change in overall prevalence and heterogeneity was caused by excluding any one study (Table 4).

**Table 4:** Summary of the leave-one-out sensitivity analysis.

Leave-one-out	Prevalence (%)	95%CI (%)	Heterogeneity	
			*I*^2^(%)	*P*
Allayed 2022	60.9	50.7–71.0	99.43	0.00
Rao 2022	61.0	50.8–71.2	99.29	0.00
García-Ayuso 2022	60.0	50.0–69.9	99.38	0.00
Salinas-Toro 2022	60.3	50.2–70.4	99.34	0.00
Abdulmannan 2022	64.7	58.0–71.5	98.53	0.00
Lin 2022	60.0	50.1–70.0	99.18	0.00
Al-Dolat 2022	59.9	50.0–69.8	99.34	0.00
Tangmonkongvoragul 2022	60.0	50.1–69.9	99.39	0.00
Long 2020	63.2	54.3–72.0	99.27	0.00
Cartes 2022	59.3	49.8–68.9	99.26	0.00
Mohan 2021	62.1	52.2–71.9	99.40	0.00

CI: Confidence interval.

### Publication bias

In the current systematic review and meta-analysis, we found no evidence of potential publication bias for the prevalence of dry eye during the COVID-19 pandemic (Egger test: B = -4.92, SE = 2.766, *P* = 0.075).

## Discussion

This is the first systematic review and meta-analysis of the prevalence of dry eye during the COVID-19 pandemic. In our study, 11 studies containing 15692 individuals were included in the analysis [17–27], which ultimately revealed a 61.0% (95%CI: 51.8%-70.2%) prevalence of dry eye during the COVID-19 pandemic. Tear Film and Ocular Surface Society (TFOS) Dry Eye Workshop (DEWS) II Epidemiology Report in 2017 stated that the global prevalence of dry eye is 5%-50% [28], and a Bayesian view suggested global prevalence of dry eye was 24.9% over the period 1997–2021 [29]. A comparison of our study with previous studies [28, 29] shows that the global prevalence of dry eye increased during the COVID-19 pandemic, which reached a staggering 61.0% (95%CI:51.8%-70.2%). Meanwhile, eight of the 11 studies included in our analysis were conducted in Asia [17, 18, 21–25, 27], and the prevalence of dry eye in Asia was estimated to be 56.7% (95%CI: 45.3%-68.1%), which is significantly higher than the 20.1% reported in a previous study [30].

During the COVID-19 pandemic, people’s lifestyle had changed dramatically, leading to psychological disorders and affecting sleep quality [31]. Not only can emotional abnormalities cause dry eye [32, 33], but there is also a correlation between sleep quality and dry eye [34–36]. For example, it has been demonstrated that lack of sleep increased tear osmolarity, reduced tear film break‑up time, and lessened tear secretions, each independently triggering dry eye [35, 37]. At the same time, obesity and nutritional imbalance caused by home diet and prolonged wearing of masks could also cause dry eye [38, 39].

It is widely believed that women’s increased susceptibility to dry eye may be related to the use of contraceptive pills and hormone production in middle-aged women, which could affect the lacrimal glands, conjunctival goblet cells, lid glands, and ocular surface sensitivity, leading to dry eye [40]. In the older-aged females, lower levels of estrogens and androgen may cause inadequate lacrimal gland secretion associated with aqueous deficient dry eye [41]. Our results showed that the prevalence of dry eye in males was 61.2% (95%CI: 58.99%-63.6%) and 69.7% (95%CI: 54.1–85.3) in females, both are significantly higher than those in previous studies before 2019 (16.7% among females and 11.4% among males) [28]. Meanwhile, our study showed a significant gender difference (OR:2.06, 95%CI: 1.47–2.88, *P*<0.001) in the prevalence of dry eye, which is line with the results of previous studies [28, 42, 43].

A previous study reported an average increase of VDT by 5 hours during the pandemic in 51.1% of their study respondents [44]. In addition, visual display causes blink rate and force changes, which could contribute to dry eye [45, 46]. Our results suggested that the OR was 2.61 (95%CI: 1.24–5.51), and there was a significant difference (*P* = 0.01) in the prevalence of dry eye between VDT of more than 4 hours per day (78.1%, 95%CI: 71.6%-84.5%) and VDT of less than 4 hours per day (58.0%, 95%CI: 43.0%-72.9%), which confirms that the prolonged use of visual display can increase the prevalence of dry eye [12–14, 28, 42, 47].

This analysis showed that the prevalence in contact lens wearers (83.5%, 95%CI: 75.7%-91.3%) was higher than in non-contact lens wearers (68.2%, 95%CI: 61.8%-71.4%), suggesting that contact lens may be a risk factor for dry eye, which is consistent with previous studies [28, 42]. Furthermore, our results were supported by a large epidemiologic study including office workers in Japan, revealing that contact lens wearers showed a 2.38 times higher risk of dry eye than non-contact lens wearers [48]. However, there was no significant difference (*P* = 0.12) between contact lens wearers and non-contact lens wearers, possibly due to the lack of included studies.

It has been shown that smoking shortens tear film break-up time, which could result in dry eye [49]. Our results indicated that the prevalence of dry eye was 74.0% (95%CI: 63.1%-85.0%) among smokers and 66.8% (95%CI: 21.4%-82.2%) among non-smokers; however, the difference was not significant (*P* = 0.12). The insufficient sample size included in the analysis and the short duration of smoking may have contributed to the lack of significant differences between smokers and non-smokers in our results.

Subgroup analysis was performed based on average age [17, 19–22, 24–27], study population [17–27], diagnostic tools [17–27], and time of study execution [17, 19–26], and heterogeneity did not change significantly. Although the prevalence varied between groups in the subgroup analyses based on average age, study population, and time of study execution, there were no significant differences (*P* = 0.649 among average age, *P* = 0.826 among study populations, and *P* = 0.344 among time of study execution).

However, in the subgroup analysis of diagnostic tools, we observed significant differences (*P* = 0.047) between the three diagnostic tools (OSDI, 64.8%, 95%CI: 55.1%-74.5%; DEQ-5, 72.6%, 95%CI: 63.0%-82.1%; and other tools, 46.6%, 95%CI: 28.1%-65.1%), which may weaken the evidence strength of other results.

The 11 studies included in our analysis were inconsistent in terms of sample size, diagnostic tools, and population characteristics; therefore, heterogeneity was considered significant, as shown by the results in Tables 3 and 4. Simultaneously, considering the differences in these studies, the results of the random-effects model, even though considered more conservative, were still used for statistical analysis to make our results more stable.

To sum up, the prevalence of dry eye during the COVID-19 pandemic is significantly higher than before, which may mean that lifestyle changes affect our eye health. In this case, protecting our eyes by changing lifestyles should be taken seriously, such as reducing visual display time, increasing outdoor activities, quitting smoking, and avoiding the wearing of contact lenses. Meanwhile, the government and medical institutions should also make more efforts to disseminate information about dry eye prevention through the Internet and media.

There are some limitations in our study. Firstly, the 11 studies included in the analysis were from 8 countries or regions in 3 continents and did not include studies performed in North America, Oceania, and Africa, leading to the possibility that the results may differ from the actual global prevalence of dry eye during the COVID-19 pandemic. Secondly, 8 of the 11 studies included in the analysis were based on school children and university students, resulting in low population representation. Finally, the 11 studies included in the analysis all used diagnostic tools, and no study using TFOS DEWS II as a diagnostic methodology, which may lead to an overestimation of the prevalence of dry eye. In addition, we found differences between diagnostic tools, which may have led to less reliable results.

Therefore, we need to include more cross-sectional surveys on the prevalence of dry eye during the COVID-19 pandemic from different countries and regions in the future. Meanwhile, studies using uniform diagnostic tools and forcing on more representative populations are also needed.

## Conclusion

The prevalence of dry eye during the COVID-19 pandemic is significantly higher than before, and a higher prevalence is found among females and those having a visual display time of more than 4 hours per day.

## Supporting information

S1 Checklist PRISMA 2020 checklist. (DOCX)

S1 Fig Forest plot of studies showing the prevalence of dry eye in terms of severity. The random-effects model was used A, B and C refer to the prevalence of dry eye in mild, moderate and severe during the COVID-19 pandemic, respectively. Pooled estimate was reported as prevalence as a percentage and CI. CI, Confidence interval.
(TIF)

S2 Fig Forest plot of studies showing the prevalence of dry eye in contact lens wearers and non-contact lens wearers. The random-effects model was used. Aand B refer to the prevalence of dry eye in contact lens wearers and non-contact lens wearers during the COVID-19 pandemic, respectively. Pooled estimate was reported as prevalence as a percentage and CI. CI, Confidence interval.
(TIF)

S3 Fig Forest plot of studies comparing the prevalence of dry eye between contact and non-contact lens wearers. The random-effects model was used. Pooled estimate was reported as odds ratio. CI, Confidence interval; IV, Inverse variance.
(EPS)

S4 Fig Forest plot of studies showing the prevalence of dry eye in smokersand non-smokers. The random-effects model was used. A and B refer to the prevalence of dry eye in smokers and non-smokers during the COVID-19 pandemic, respectively. Pooled estimate was reported as prevalence as a percentage and CI. CI, Confidence interval.
(TIF)

S5 Fig Forest plot of studies comparing the prevalence of dry eye between smokers and non-smokers. The random-effects model was used. Pooled estimate was reported as odds ratio. CI, Confidence interval; IV, Inverse variance.
(EPS)

S6 Fig Forest plot of studies showing subgroup analysis of study population. The random-effects model was used. Pooled estimate was reported as prevalence as a percentage and CI.CI, Confidence interval.
(TIF)

S7 Fig Forest plot of studies showing subgroup analysis of average age. The random-effects model was used. Pooled estimate was reported as prevalence as a percentage and CI. CI, Confidence interval.
(TIF)

S8 Fig Forest plot of studies showing subgroup analysis based on time of study execution. The random-effects model was used. Pooled estimate was reported as prevalence as a percentage and CI. CI, Confidence interval.
(TIF)
