# Supplementary figures and images for: Prevalence of dry eye during the COVID-19 pandemic: A systematic review and meta-analysis
Source: PLoS One. 2023 Dec 13;18(12):e0288523. doi: 10.1371/journal.pone.0288523 (PMC10718424; doi:10.1371/journal.pone.0288523)

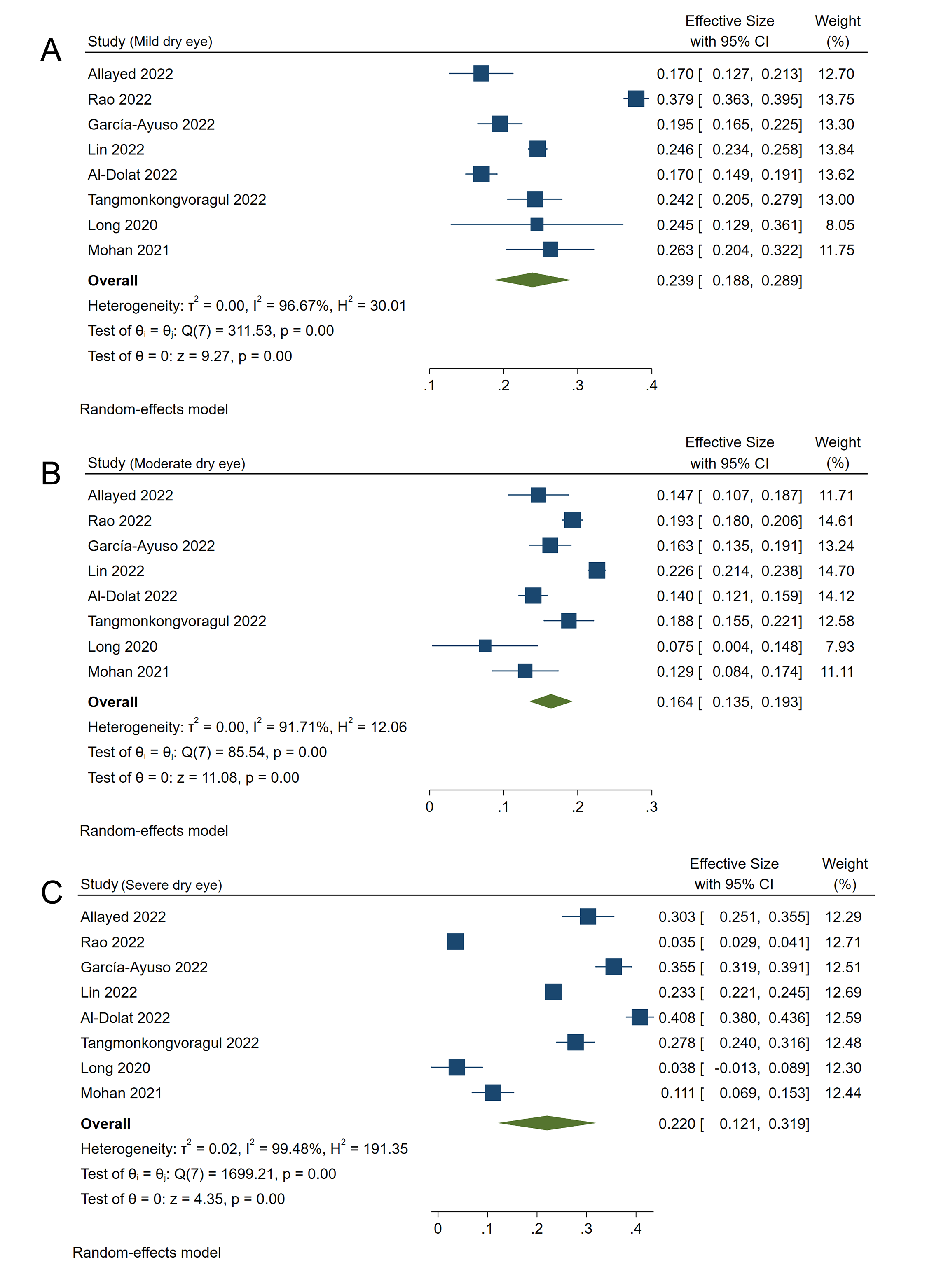

Supplement: S1 Fig — The random-effects model was used A, B and C refer to the prevalence of dry eye in mild, moderate and severe during the COVID-19 pandemic, respectively. Pooled estimate was reported as prevalence as a percentage and CI. CI, Confidence interval. (TIF) [file pone.0288523.s002.tif]

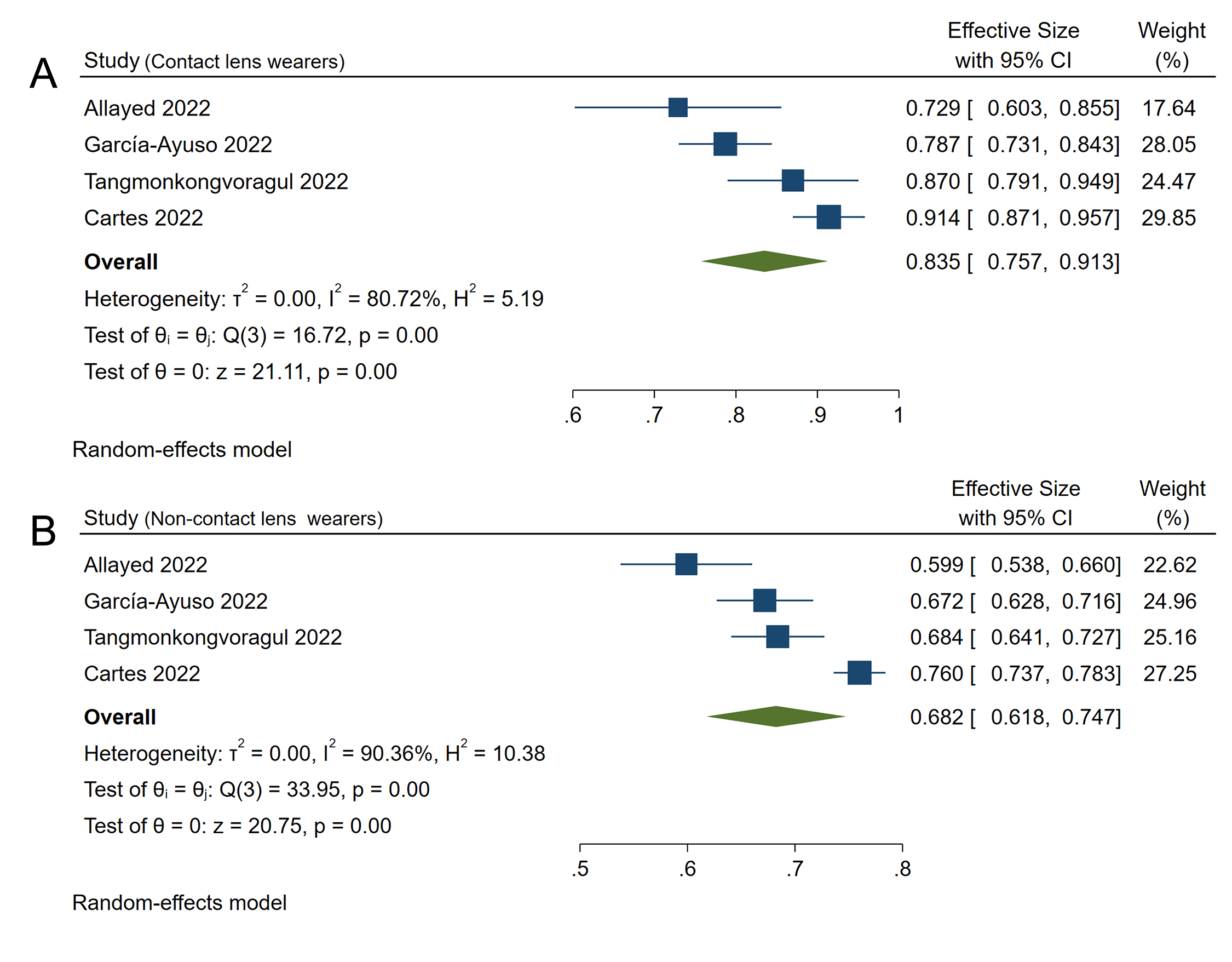

Supplement: S2 Fig — The random-effects model was used. Aand B refer to the prevalence of dry eye in contact lens wearers and non-contact lens wearers during the COVID-19 pandemic, respectively. Pooled estimate was reported as prevalence as a percentage and CI. CI, Confidence interval. (TIF) [file pone.0288523.s003.tif]

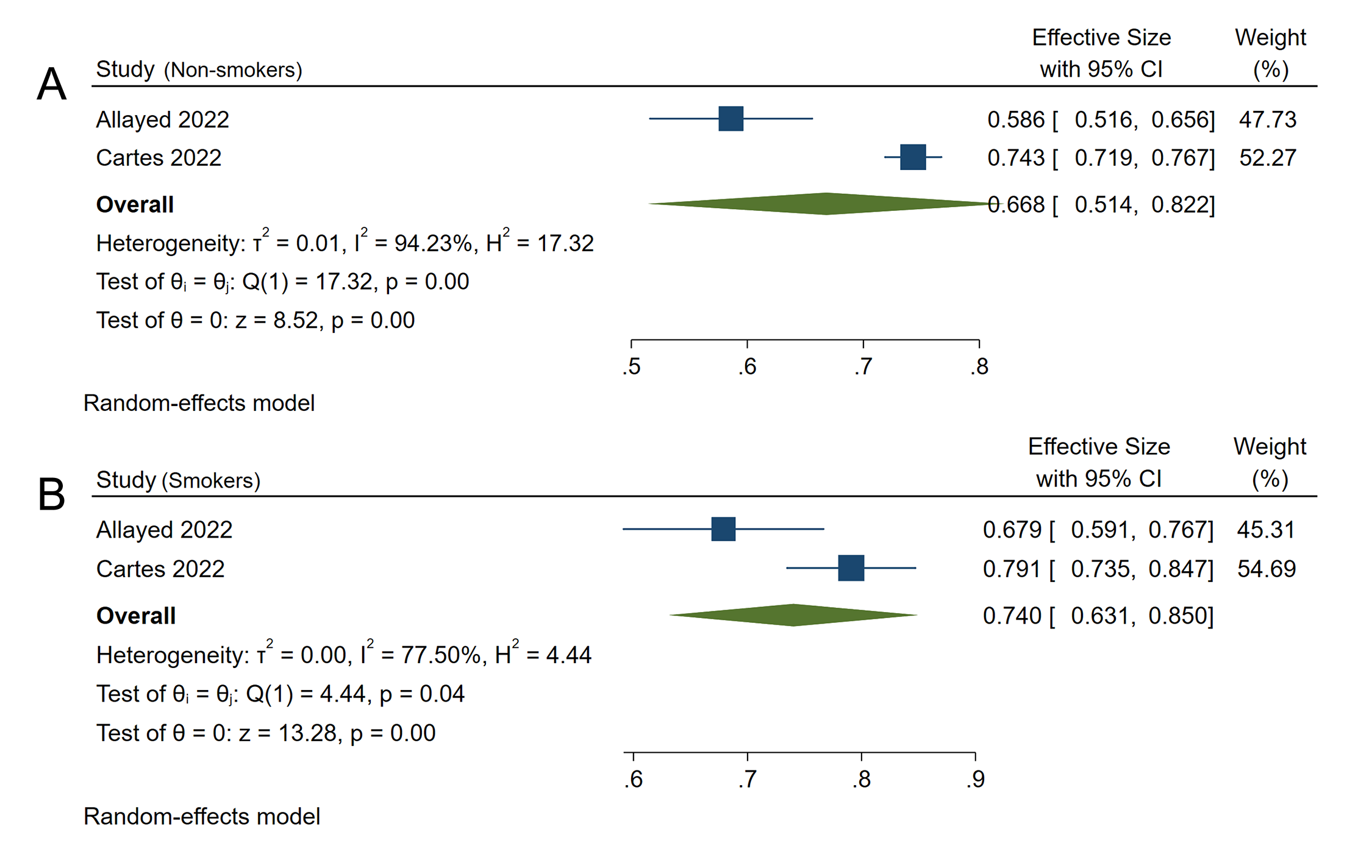

Supplement: S4 Fig — The random-effects model was used. A and B refer to the prevalence of dry eye in smokers and non-smokers during the COVID-19 pandemic, respectively. Pooled estimate was reported as prevalence as a percentage and CI. CI, Confidence interval. (TIF) [file pone.0288523.s005.tif]

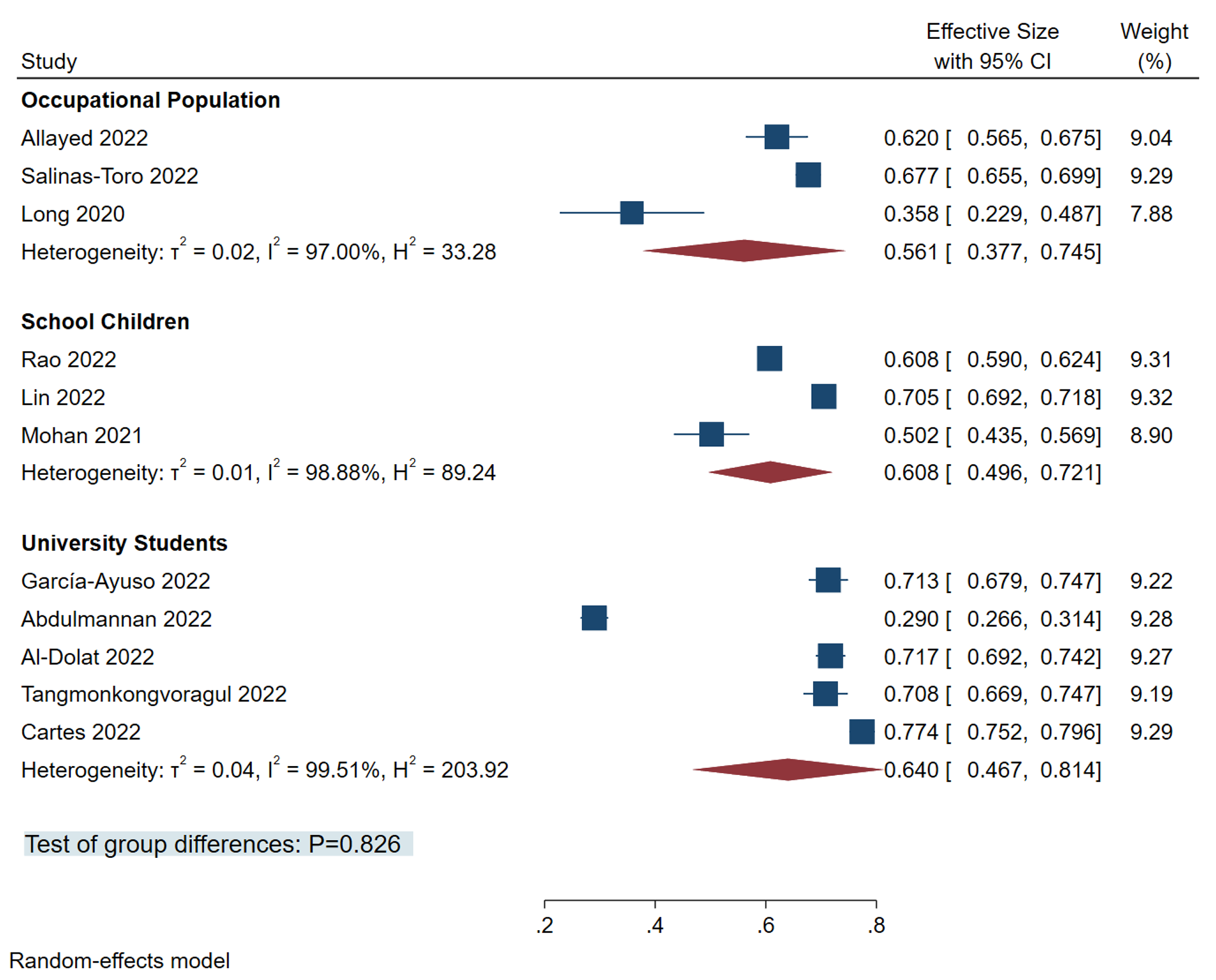

Supplement: S6 Fig — The random-effects model was used. Pooled estimate was reported as prevalence as a percentage and CI.CI, Confidence interval. (TIF) [file pone.0288523.s007.tif]

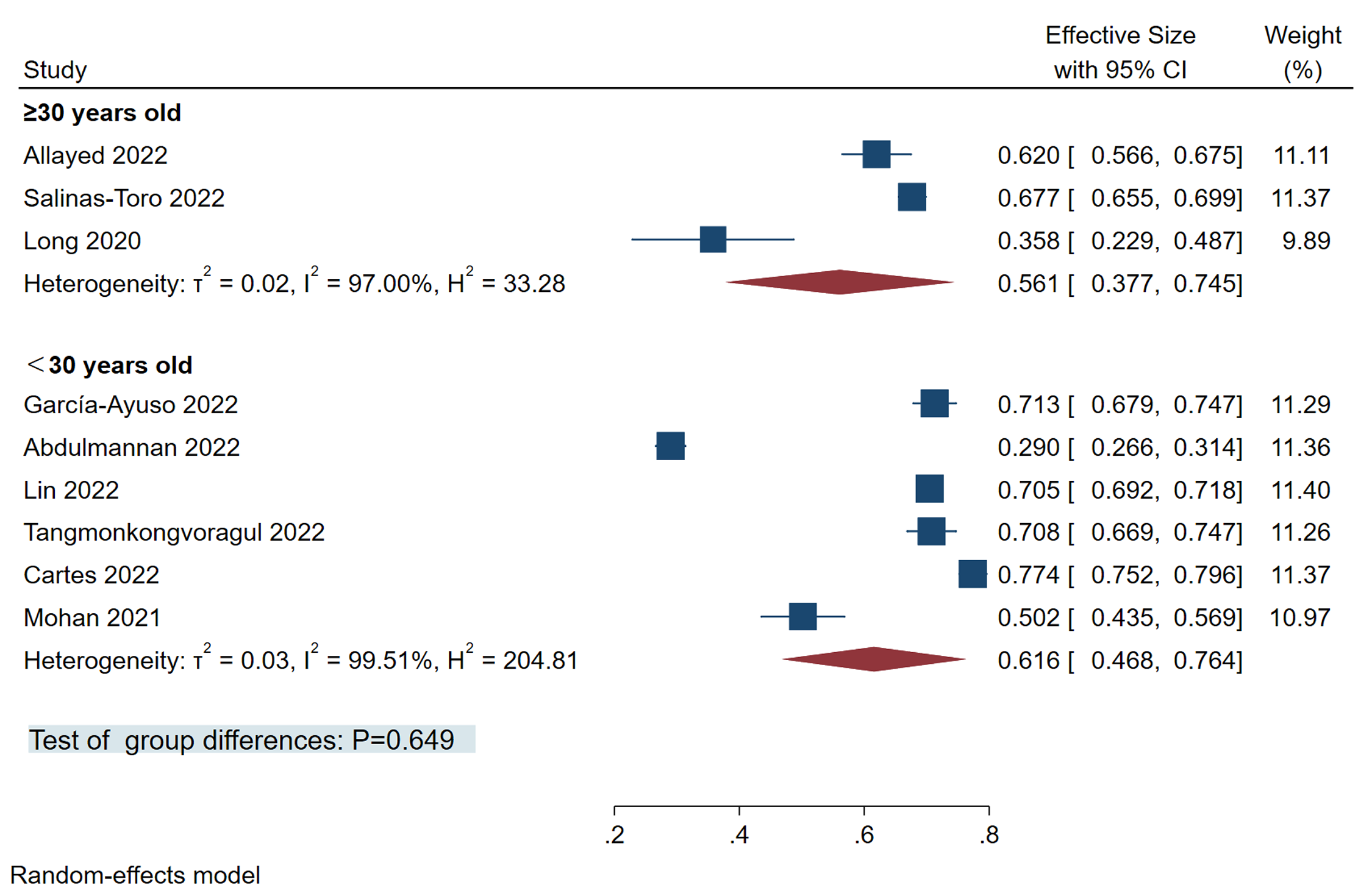

Supplement: S7 Fig — The random-effects model was used. Pooled estimate was reported as prevalence as a percentage and CI. CI, Confidence interval. (TIF) [file pone.0288523.s008.tif]

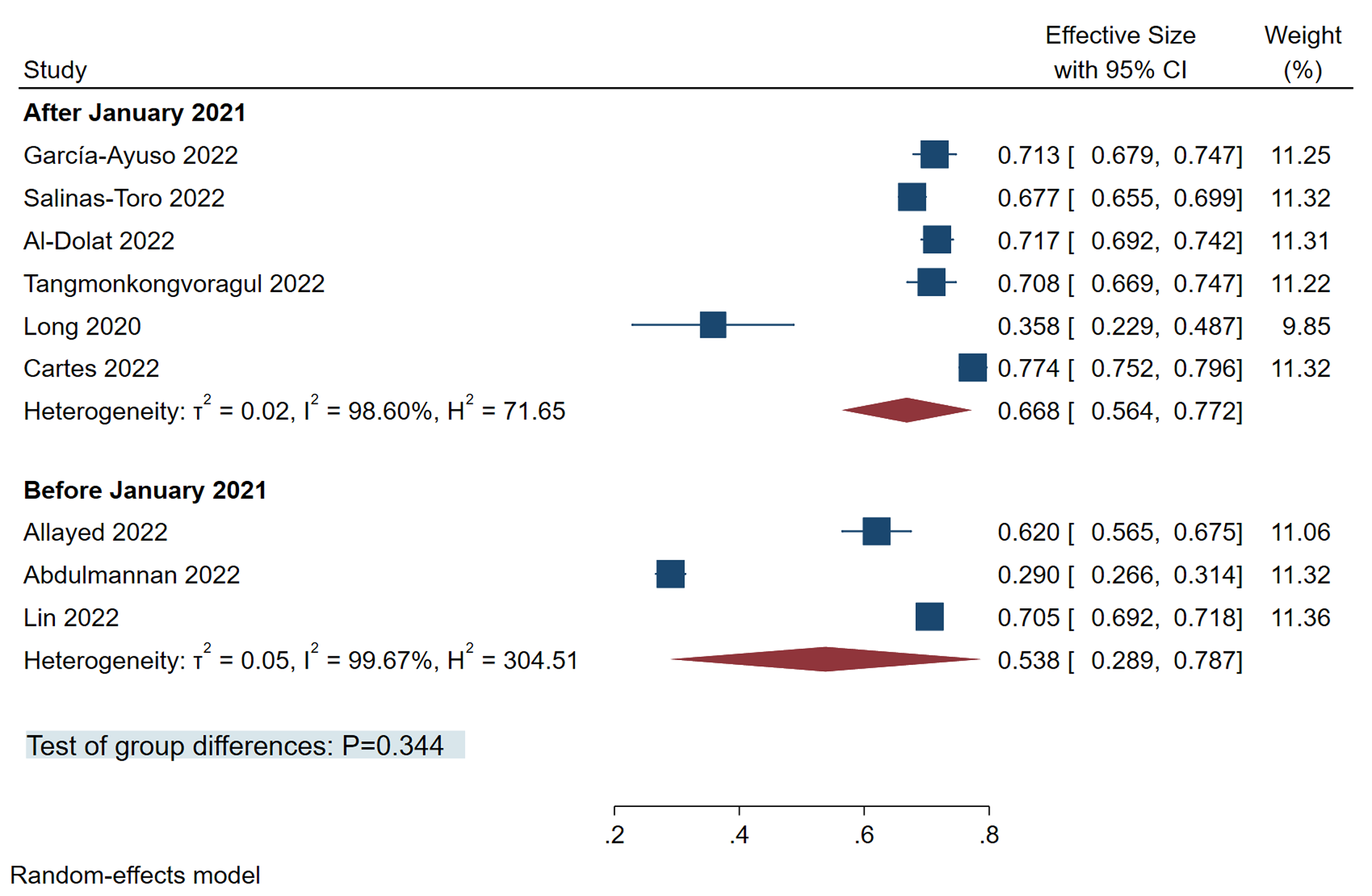

Supplement: S8 Fig — The random-effects model was used. Pooled estimate was reported as prevalence as a percentage and CI. CI, Confidence interval. (TIF) [file pone.0288523.s009.tif]
